# Clinical evaluation of MiADE: a natural language processing system for assisting structured diagnosis recording at the point of care

**DOI:** 10.1136/bmjhci-2025-101801

**Published:** 2026-02-11

**Authors:** Mairead McErlean, Jack Ross, Jonathan Kossoff, Maisarah Amran, James Brandreth, Leilei Zhu, Gary Philippo, Wai Keong Wong, Folkert W Asselbergs, Richard J B Dobson, Yogini Jani, Enrico Costanza, Anoop Dinesh Shah

**Affiliations:** 1 Centre for Medicines Optimisation Research & Education, University College London Hospitals NHS Foundation Trust, London, UK; 2 Department of Clinical Pharmacology, University College London Hospitals NHS Foundation Trust, London, UK; 3 Department of Acute Medicine, University College London Hospitals NHS Foundation Trust, London, UK; 4 Institute of Health Informatics, UCL, London, UK; 5 National Institute for Health and Care Research University College London Hospitals Biomedical Research Centre, University College London Hospitals NHS Foundation Trust, London, UK; 6 Clinical Coding Services, Guy's and St Thomas’ Hospitals NHS Trust, London, UK; 7 University College London Hospitals NHS Foundation Trust, London, UK; 8 Cambridge University Hospitals NHS Foundation Trust, Cambridge, UK; 9 Department of Cardiology, Amsterdam University Medical Centres, Amsterdam, The Netherlands; 10 Department of Biostatistics and Health Informatics, King’s College London Institute of Psychiatry Psychology & Neuroscience, London, UK; 11 School of Pharmacy, University College London, London, UK; 12 UCL Interaction Centre, UCL, London, UK

**Keywords:** Medical Records Systems, Computerized, Medical Informatics, Documentation

## Abstract

**Objectives:**

To evaluate the usability, usefulness and impact of a novel point of care natural language processing (NLP) system, Medical information AI Data Extractor (MiADE), to assist structured diagnosis recording in electronic health records.

**Methods:**

Mixed methods evaluation of the implementation of MiADE in a major National Health Service hospital, with surveys, interviews and observed outpatient consultations. The number of structured diagnoses recorded per outpatient encounter was compared before and after MiADE, and completeness of inpatient problem lists was evaluated using billing diagnoses as a gold standard.

**Results:**

85 clinicians consented to the study and were provided access to MiADE and 24 used MiADE to receive structured data suggestions during the study period. Baseline survey data and observations showed wide variation in structured data recording despite clinicians considering it to be important. Half of postimplementation survey respondents considered MiADE to be ‘very’ or ‘moderately’ useful. Multilevel quasi-Poisson regression of 12 309 outpatient encounters (accounting for time and clustering by clinician) estimated that the post-MiADE period was associated with 23.7% more diagnoses recorded per encounter. No improvement was seen in the inpatient setting.

**Discussion:**

Structured recording of key information such as diagnoses using a clinical terminology is essential for safe, efficient patient care, but is currently done incompletely because it is time-consuming for clinicians. MiADE was associated with an increase in outpatient structured diagnosis recording despite low uptake of the tool.

**Conclusion:**

Point of care NLP using MiADE can potentially improve structured data recording, but further development and better clinician engagement are needed to maximise its impact.

**Trial registration number:**

ISRCTN58300671.

**WHAT IS ALREADY KNOWN ON THIS TOPIC**

- Despite the benefits of structured recording of healthcare data for individual care and research, much of the key information in electronic health records (such as diagnoses) is only recorded in free text.
- Structured data entry in electronic health record systems can be time-consuming and cumbersome for clinicians.

**WHAT THIS STUDY ADDS**

- Embedding natural language processing within the electronic health record to suggest structured data entries was reported by clinicians to be useful and increased the recording of outpatient diagnoses.
- Clinician engagement was challenging, and overall usage of structured data remained suboptimal.

**HOW THIS STUDY MIGHT AFFECT RESEARCH, PRACTICE OR POLICY**

- Usability of electronic health record systems needs to improve to enable clinicians to record high-quality data without impeding their workflow.
- Natural language processing embedded within electronic health records may improve ease of use for data entry, but high-level clinician buy-in is also needed to influence professional documentation practice.

## Introduction

 Structured recording of key information such as diagnoses in electronic health records (EHR) improves safety^1^ and is recommended in professional guidance^2 3^ but is currently incompletely done^4^ because it can be onerous for clinicians to enter structured data.^5^,^7^

Previous work to improve structured data recording has shown that tools are more likely to be successful if fully embedded in the workflow.^8^ Natural language processing (NLP)^9^ has been suggested as a possible solution, and commercial systems for point of care Systemized Nomenclature of Medicine – Clinical Terms (SNOMED CT) coding are available,^10 11^ but there is little published data on their effectiveness and usability in the clinical setting. Kaufman *et al* compared NLP and standard documentation methods in a simulation setting.^12^ Blasco *et al* implemented an NLP-assisted point of care SNOMED CT coding system in Barcelona, but did not include a qualitative assessment.^13^

We, therefore, developed, implemented and evaluated a system called Medical information AI Data Extractor (MiADE) in a major National Health Service (NHS) hospital. MiADE embeds NLP within the EHR user interface to assist structured entry of diagnoses.^14^ The aims of our study were to: (1) capture clinician views on data documentation processes, (2) evaluate the usability, usefulness and impact of MiADE for recording diagnoses and (3) develop recommendations to maximise the effectiveness of such systems.

## Methods

This was a mixed-method, single-centre before-and-after study. This study is registered on the ISRCTN registry (ISRCTN58300671).^15^

### Structured diagnosis recording in the EHR

The EHR system used at University College London Hospitals (UCLH), Epic, enables clinicians to enter SNOMED CT-coded problem list and encounter diagnoses,^16^ which typically involves keyword searching or browsing the SNOMED CT hierarchy to find the appropriate concept.

**Figure 1:**
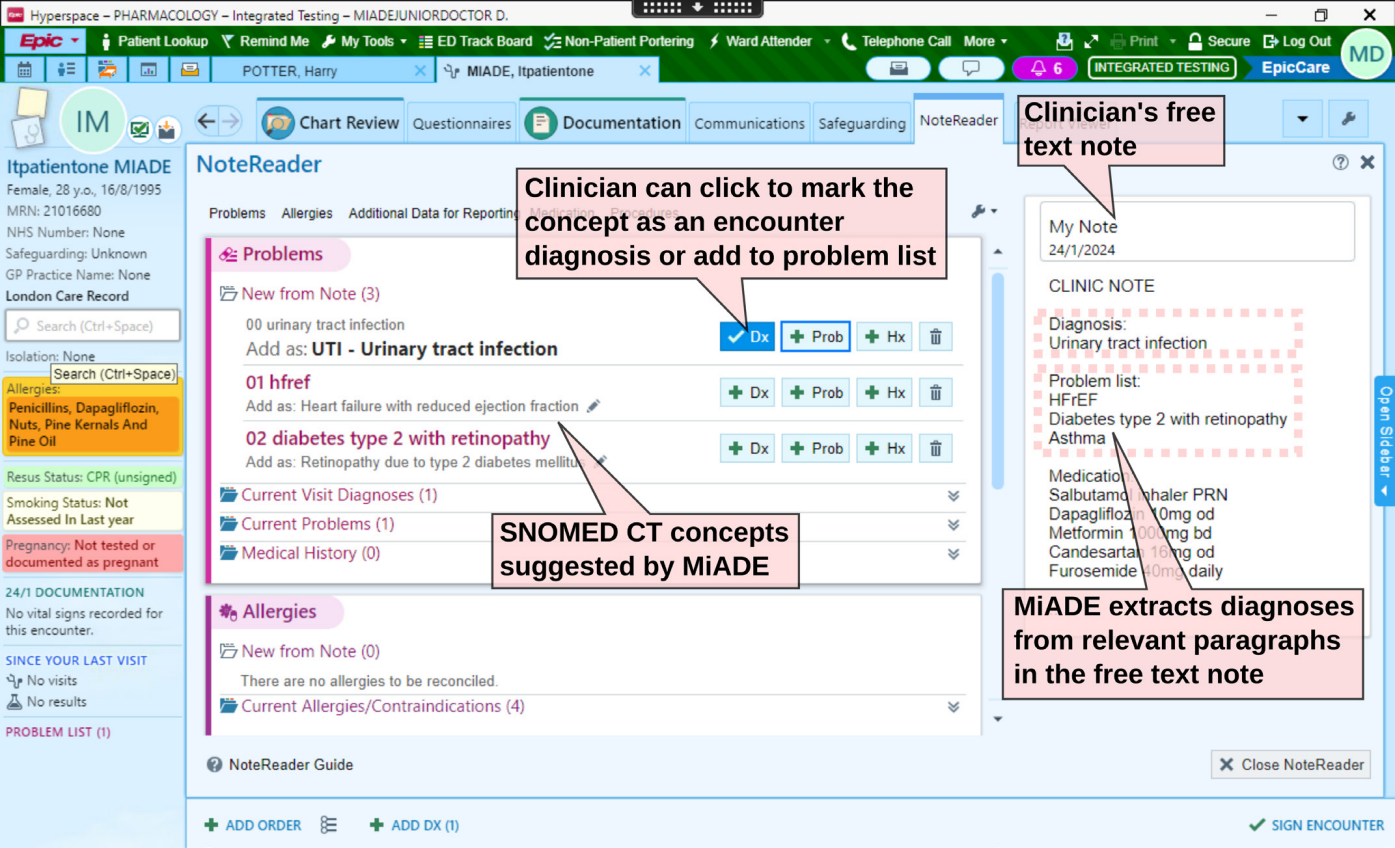
Screenshot of the Epic NoteReader electronic health record interface, which displays the clinical note and MiADE suggestions side by side. The clinician can add the SNOMED CT concepts suggested by MiADE to the problem list with a few clicks. The MiADE suggestions are prefixed by numbers to ensure that NoteReader displays them in the correct order. Copyright Epic Systems Corporation. MiADE, Medical information AI Data Extractor. SNOMED CT, Systemized Nomenclature of Medicine – Clinical Terms. NHS, National Health Service. HFrEF, heart failure with reduced ejection fraction. PRN, as required.

MiADE provides an alternative way to enter SNOMED CT concepts, via the Epic ‘NoteReader’ interface, which displays automatic SNOMED CT concept suggestions alongside the original text when saving a note (figure 1). The clinician can then add the relevant entries with just a few clicks. If MiADE is available for a user, they will see a ‘NoteReader’ toggle button on their screen, which they can use to invoke MiADE whenever they save a note. MiADE is non-interruptive; clinicians can ignore it and continue their usual workflow if they wish.

### MiADE design and implementation

MiADE was built using the MedCAT open-source NLP system^17^ and underwent testing and Trust safety case approval before live implementation.^14^ The evaluation study was advertised to clinicians by email, intranet blogs and presentations at clinical meetings, and opened for recruitment on 27 December 2023. Participants were asked to watch a 5 min training video and complete an online survey and consent form. A focus group was held 1 month after go-live, following which changes were made to the filtering of MiADE suggestions. MiADE was originally configured to return diagnoses and other clinical findings from paragraphs with a relevant header (eg,‘Problem list’) and up to one concept from elsewhere in the text. The change was to filter out non-diagnosis concepts (eg, symptoms) and only analyse paragraphs with a relevant header.

### Study procedures

Individual clinician surveys were distributed before and after MiADE implementation using the Qualtrics XM platform, with minimum 2-week usage required before completing the second survey. Observations of outpatient clinics were conducted in the same timeframe, aiming to observe at least five clinicians in both phases, with purposive sampling to include a range of specialties and seniorities (online supplemental figure 1). Postimplementation, at least five inpatient clinicians participated in one-to-one semistructured interviews via MS Teams or telephone (online supplemental material 1: interview topic guide). Four MiADE investigators were hospital clinicians at the study site and were excluded from analysis.

### Qualitative data analysis

Audio transcripts of clinician and patient interviews were collected on a secure digital voice recorder and transcribed by a professional service. The interviews were 10–20 min in duration. The clinician workshop was recorded and transcribed via MS Teams. Observations and field notes were recorded by one researcher (MM, a female research pharmacist) using a rapid research evaluation form designed by the research team. Recorded transcripts and text files were stored securely on trusted NHS network servers.

MAXQDA Plus 2022 (VERBI Software) was used to analyse the raw data transcripts. One author, (MM), coded the initial transcripts and summarised the findings. Cross-validation of coding and analysis was independently performed by two authors, an experienced female doctor of pharmacy (YJ) and a male professor of human-computer interaction (EC). The final codebook was agreed by all authors. Thematic analysis was undertaken using inductive and deductive approaches, ensuring that thematic saturation was achieved and was informed by the Technology Acceptance Model^18^ and the Theoretical Domains Framework.^19^

### Quantitative data analysis

We extracted diagnosis records and data on MiADE usage from the hospital’s data warehouse to compare diagnosis recording before MiADE go-live (1 November 2023 to 25 February 2024) and after MiADE go-live (26 February 2024 to 25 February 2025). Patients who opted out of data use for research and those under the care of MiADE investigators were excluded.

For inpatients under the care of participating teams, we calculated the completeness of problem lists on discharge for the first admission of each patient during the study period, considering the ICD-10 billing diagnoses to be the gold standard. We considered an ICD-10 code to be included if an SNOMED CT problem mapping to any ICD-10 code in the same block was present on or prior to the discharge date. We carried out a sensitivity analysis requiring a stricter match (same three-character ICD-10 code).

We assessed outpatient use of MiADE by calculating the number of encounter diagnoses and problem list entries made per encounter within a week of the clinic date, for the earliest clinic encounter per patient within the study period. We compared time periods before and after MiADE, using a multilevel quasi-Poisson regression with random effects for clinician clustering and a time variable.

## Results

Eighty-five clinicians working in a range of specialties (27 consultants, 52 resident doctors, 1 dentist, 2 general practitioners, 1 physician assistant, 1 advanced nurse practitioner and 1 midwife) consented to the study (online supplemental table 1). Eight clinicians attended the postimplementation focus group, and the median duration of access to MiADE was 270 days.

Fifty-six clinicians completed the pre-MiADE questionnaire and seven were observed in outpatient clinics, of whom five were reobserved post-MiADE. We also interviewed 16 patients (10 pre-MiADE and 6 post-MiADE) and 7 inpatient clinicians (online supplemental figure 1). Twelve clinicians completed the post-MiADE questionnaire. Full anonymised survey results are in online supplemental material 2: full survey results.

### Clinician documentation practices

**Figure 2:**
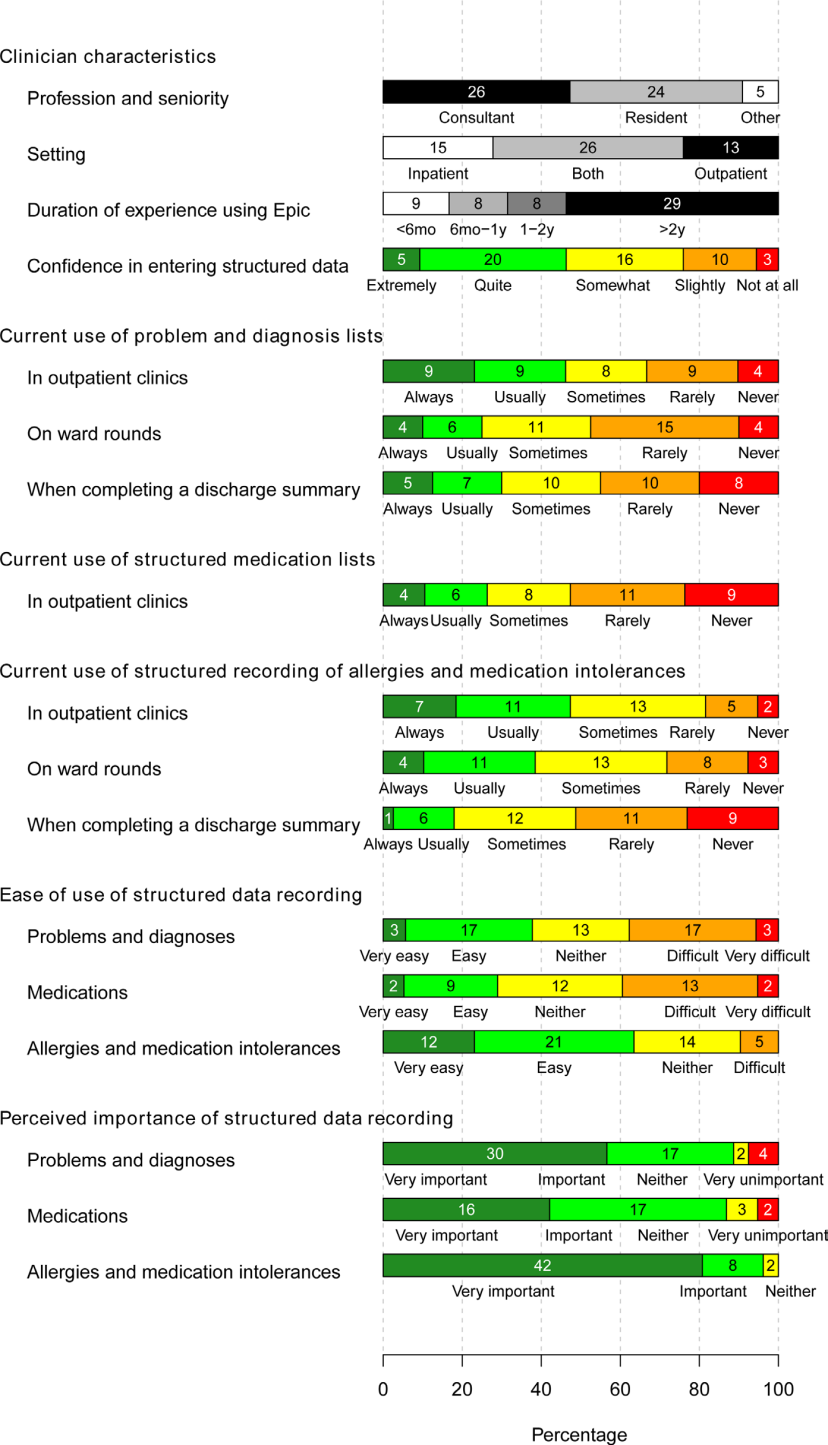
Results of pre-MiADE survey (56 responses). MiADE, Medical information AI Data Extractor.

Questionnaire responses suggested that under half of clinicians (18/39) ‘always’ or ‘usually’ use problem lists in outpatient clinics, and a quarter (10/40) do so on inpatient ward rounds (figure 2). Clinic observations corroborated this finding, with all clinicians preferentially completing documentation using free text. The freedom of choice in documentation created variation in how the information was stored, and burdened clinicians with gathering relevant data from multiple locations in a patient’s record (focus group; table 1, quotes 1–4).

**Table 1:** Quotes on baseline documentation practices in electronic health records which support themes identified in qualitative analysis

Quote number	Quote
1	‘The amount of time every doctor spends looking at the notes to read what’s wrong with the patient is enormous’. (Focus Group)
2	‘Lists can contain too much information which makes it difficult to read/ process quickly’. (Q019, Consultant, Care of the Elderly, Pre-MiADE Survey)
3	‘(Structured data): Not generally used on ward in my experience, sometimes (particularly in elder patients) oversaturated with information, much of which is less relevant and difficult to fully digest’ (Q041, FY1 Doctor, Infectious Diseases, Pre-MiADE Survey)
4	‘…the ideal world …is when you click on epic, you open a patient, you look at the problem list. It is the best place to tell you immediately what is wrong with the patient. In order to do that you need to be able to distinguish between what is your active hospital problem and what is your past medical history so those bits have to be separated off. Because if you don't understand that narrative, it’s completely useless and senseless and it’s just a big pile of stuff’. (Focus Group)

MiADE, Medical information AI Data Extractor.

Clinicians also expressed confusion about when to enter structured data, whether it should be entered at initial presentation or after confirming a diagnosis (outpatient clinic observations).

### Barriers and enablers of structured data entry

We identified several factors influencing structured data entry: clinicians' perceptions of value, time and workload constraints, knowledge of the EHR system, team culture and the interface usability.

#### Clinician beliefs

Survey data revealed that clinicians recognised the value of structured data entry (figure 2), with over 85% of respondents rating the structured recording of problems, medications and allergies as ‘very important’ or ‘important’. Respondents mentioned numerous benefits including enhanced patient safety, improved care co-ordination, streamlined administrative processes and supporting research. However, adoption of structured data entry was inconsistent (online supplemental table 2, quote 5).

Structured documentation for medication and allergies was considered important and easier to enter in the EHR than problem lists (online supplemental table 2, quotes 6–7).

Clinician concerns about structured documentation included data quality, maintenance and overall usability. They voiced a lack of trust in problem lists, citing perceived inaccuracies and incompleteness. As a result, they often disregarded structured documentation and relied on free-text notes, creating a negative feedback loop (online supplemental table 2, quotes 8–9).

Many clinicians preferred using free text to capture the nuance of patient consultations (online supplemental table 2, quote 10). They said free text provides ‘lots of opportunity to add detail’ and allows them to describe how conditions ‘…affect the patient’ (online supplemental table 2, quotes 11–12). Additionally, some clinicians preferred free-text documentation as a permanent entry that cannot be altered by others (online supplemental table 2, quote 13).

Many clinicians did not perceive structured documentation as a core responsibility of their role (online supplemental table 2, quotes 14–15).

#### Baseline knowledge and training

A shared understanding of EHR functionality and terminology is crucial to ensure quality of documentation. Some clinicians mentioned their teams organised individual sessions in the absence of central training on EHR documentation best practices (online supplemental table 2, quote 16).

#### Workflow and time constraints

EHR documentation is often time-consuming, especially for complex or new patients (outpatient clinic observations). Clinicians reported that navigating multiple screens to review and update notes was cumbersome and inefficient, making it harder to access key patient information (online supplemental table 2, quotes 17–18).

Additionally, clinician workload and the demands of the clinical setting significantly impact the time available for documentation (online supplemental table 2, quote 19).

#### Team culture and practices

Some teams adopted strategies to encourage the use of structured data, such as templates that pull structured documentation into progress notes, providing an organised framework, and using reminders to update the problem list before discharge. In other teams, structured documentation was not prioritised, and engagement was low (online supplemental table 2, quote 20). Furthermore, there was recognition that inconsistent documentation practices across different teams involved in a patient’s care contributed to challenges with data quality (online supplemental table 2, quote 21).

#### EHR usability

Survey responses showed a wide range of opinions regarding ease of structured data entry (figure 2). The existing interface design required clinicians to switch between screens frequently, disrupting their workflow (online supplemental table 2, quotes 22–24). Clinicians also mentioned it was confusing for infrequent users as and when the system interface changed (online supplemental table 2, quotes 25–26).

Features that aid structured documentation, such as access via a non-intrusive sidebar or reminder prompts (as for allergies and medications), were considered by some to be easier to use than the interface for entering problems (online supplemental table 2, quotes 27–28). Dislike of the aesthetic of structured documentation negatively impacts its use (online supplemental table 2, quote 29).

System usability in modifying existing entries such as medication dosages or allergy statuses and having to search for the appropriate codes or understand the terminology for their entries also had a negative influence (online supplemental table 2, quotes 30–32).

### Experience using MiADE

#### Perceived usefulness

**Figure 3:**
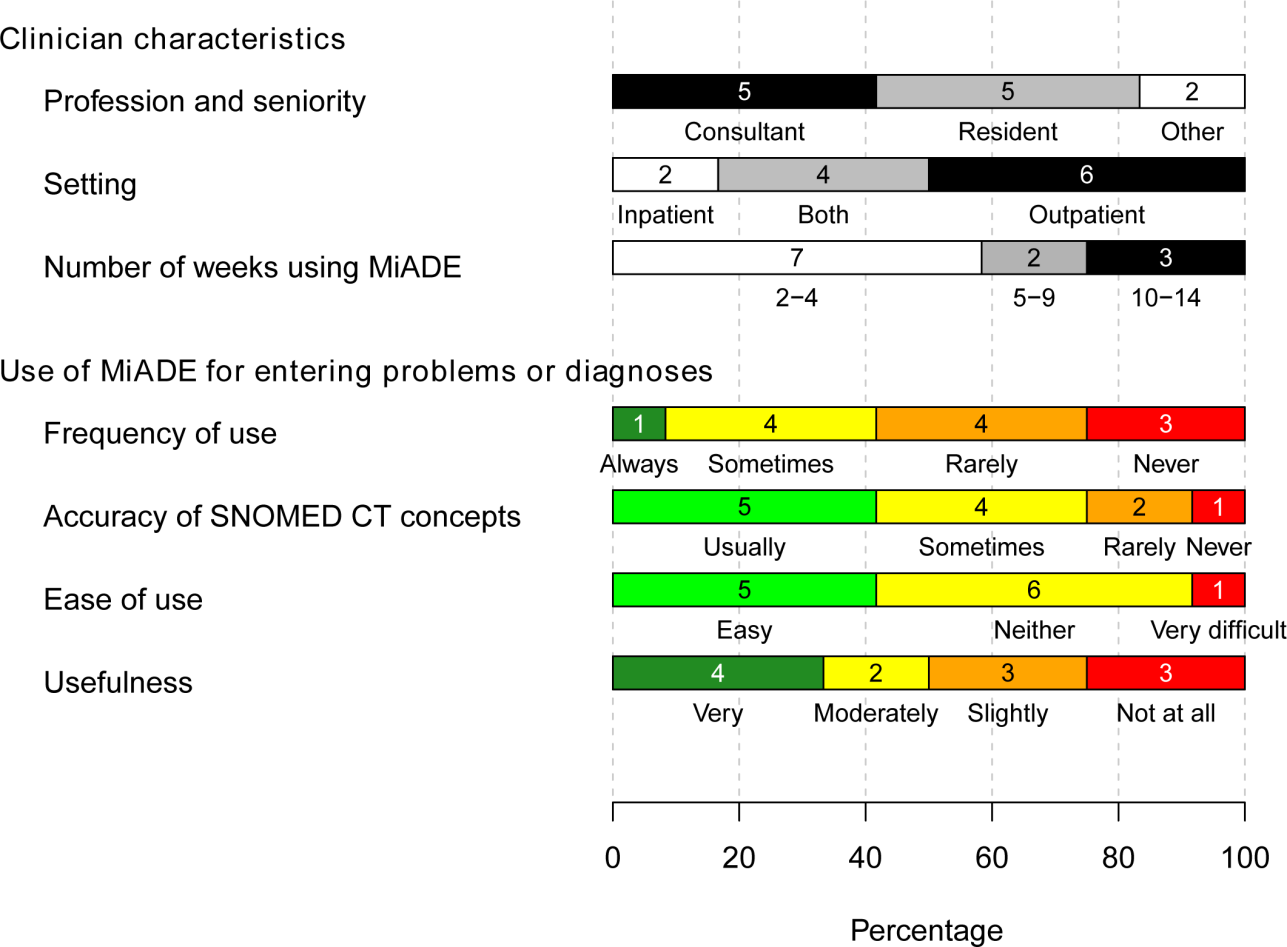
Results of post-MiADE survey (12 responses). MiADE, Medical information AI Data Extractor. SNOMED CT, Systemized Nomenclature of Medicine – Clinical Terms.

Half of the survey respondents considered MiADE to be ‘very’ or ‘moderately’ useful (figure 3). For clinicians who infrequently entered structured information, MiADE simplified the process by automating data entry and reducing manual effort (online supplemental table 3, quotes 33–36). Some clinicians who generally enter structured information only for their specialty commented that MiADE prompted them to add other conditions, which they usually would not do (online supplemental table 3, quote 37).

Despite streamlining some aspects, clinicians felt that MiADE introduced additional time to the overall documentation process (online supplemental table 3, quotes 38–41) and could sometimes be slow and get ‘stuck’ (Post-MiADE outpatient clinic observations).

#### Accuracy and trust

Clinicians reported that MiADE sometimes returned inaccurate suggestions or failed to recognise relevant diagnoses. These inaccuracies, particularly false positives, were reported to potentially erode clinician trust and discourage the use of MiADE (online supplemental table 3, quotes 42–45).

#### Engagement and training

Many clinicians did not recall undertaking the MiADE training, leading to confusion and suboptimal usage. Despite signing up to the study, some clinicians were unaware that MiADE was available to use (Field Notes, Post-MiADE Outpatient Clinic Observations; online supplemental table 3, quote 46).

Engagement and acceptability of MiADE were strongly linked to the clinician’s usual workflow. Those clinicians who already used structured documentation on EHR preferred to continue with that whereas those who did not enter structured information before found MiADE more beneficial (online supplemental table 3, quotes 47–48).

#### System improvement suggestions

Several clinicians stated more automation of coding would improve structured documentation; however, only one referred to the process of actively reviewing the MiADE suggestions as an important step to minimise the risk of incorrect entries being added to the patients’ record (online supplemental table 3, quotes 49–50).

### Patient perspectives on documentation

Patients confirmed that they were satisfied with their doctor’s use of the computer during their consultation. Some patients did not notice the clinician using the computer and were more concerned with the outcome of the consultation, whereas others felt reassured that their doctor was documenting what they were saying during the consultation and liked when the clinician used the computer as an aid in the consultation.

Patients did not seem to be aware of the potential uses or benefits of structured information within the EHR and did not notice any difference in the consultation process following the implementation of MiADE.

### Impact of MiADE on structured data recording

Forty of the 85 participating clinicians carried out at least one outpatient consultation during the study period (online supplemental figure 2). Among the 12 309 outpatient consultations analysed, we found a crude 36% increase in the mean number of problems or diagnoses per encounter from 0.665 to 0.904 after MiADE go-live (increase of 0.239; 95% CI 0.194 to 0.284; p<0.001 by t-test) (table 2). However, only 14 outpatient clinicians actually received MiADE suggestions during the study period. Among this group, there was also an increase in the proportion of encounters with at least one problem or diagnosis entered, from 49.6% to 54.0% (p<0.001 by proportion test) (table 2).

**Table 2:** Recording of structured diagnoses in outpatient encounters before and after MiADE go-live, by clinician’s MiADE activation status

Time period	Outpatient clinicians actually using MiADE (n=14)		Outpatient clinicians with MiADE available but not using (n=26)	
	Pre-MiADE	Post-MiADE	Pre-MiADE	Post-MiADE
Number of patients	1342	3408	2639	4920
Median age in years	43	44	48	46.5
Percentage (N) female	62.9% (844)	59.3% (2022)	58.4% (1541)	56.6% (2784)
Total number of diagnoses	1081	3525	1567	4001
Number (%) of diagnoses entered using MiADE	0	429 (12.2%)	0	0
MiADE acceptance rate with original filter (before 15 April 2024)	–	21.6% (155/719)	–	–
MiADE acceptance rate with amended filter (after 15 April 2024; only diagnoses under a relevant heading)	–	77.0% (274/356)	–	–
Number of encounters with at least one diagnosis	666	1842	1081	1972
Percentage (95% CI) of encounters with at least one diagnosis	49.6% (46.9% to 52.3%)	54.0% (52.4% to 55.7%)	41.0% (39.1% to 42.9%)	40.1% (38.7% to 41.5%)
Mean (SD) number of diagnoses per encounter	0.806 (1.11)	1.03 (1.47)	0.60 (0.95)	0.81 (1.51)

‘Diagnoses‘ refers to encounter diagnoses or problem list entries created during or within one week of the consultation. Each patient was included only once per time period.

MiADE, Medical information AI Data Extractor.

Analysis by multilevel quasi-Poisson regression with random effects for clinician clustering and a time variable showed that the period after MiADE go-live was associated with 23.7% more diagnoses per encounter (95% CI 1.144 to 1.337, p<0.001). There was no statistically significant difference in diagnosis recording by MiADE filter status (p for interaction=0.082) or whether the clinician was actually using MiADE (p for interaction=0.659) (online supplemental table 4).

Among inpatient teams, there was no significant difference in the number of diagnoses recorded per patient before and after MiADE go-live, but the post-MiADE period was associated with a lower proportion of ICD-10 codes with a corresponding problem list item (113/1263 before vs 170/3336 after, p<0.001 by proportion test) (online supplemental table 5). There was a variable level of engagement with MiADE among inpatient teams, with few users consented and very little problem list usage in some teams (online supplemental table 6).

The acceptance rate of MiADE suggestions improved from 22.6% (192/850) to 73.6% (309/420, p<0.001 from proportion test) after amending the filtering of MiADE suggestions to return only diagnoses under a relevant heading (online supplemental table 1). MiADE usage was highest during the first 5 weeks of the study (mean 5.6 users per week) and then dropped to a low level, with mean 1.4 users per week from week 6 onwards (online supplemental figure 3). No patient safety incidents related to MiADE were reported during the study. Mean note processing time of MiADE was 3.25 s per document.

## Discussion

### Key results

This study demonstrates the safe deployment of an NLP-based structured documentation tool (MiADE) within a live clinical EHR environment. The study also highlights the challenges in engaging with clinicians to influence EHR use within their busy workload. Fewer than a third of signed-up participants activated MiADE and received NLP suggestions, but nevertheless the time period following MiADE go-live was associated with 24% more diagnoses recorded per outpatient encounter. Use of MiADE varied considerably between clinicians, influenced by individual preferences, specialty-specific needs and familiarity with structured documentation.

Qualitative findings demonstrate the persistent challenges in embedding structured data entry into clinical workflows. While clinicians appreciate the importance of structured documentation, there was uncertainty about when and by whom structured information should be entered, and significant baseline variation in use of structured data in the EHR.

The lack of effectiveness of MiADE in the inpatient setting may have been due to lack of a critical mass of users within each department, lack of a culture of structured data recording and staff turnover (the majority of inpatient users were resident doctors who rotate every 4–6 months).

Time constraints and workflow disruptions were consistently cited as a barrier to high-quality documentation, consistent with previous studies.^20^,^22^ Research on EHR usability has found that systems often interrupt clinical workflows, add to clinicians’ cognitive load and contribute to note bloat, clinician burnout and reduced patient interaction time.^23^,^25^

Clinicians reported that MiADE was a helpful adjunct to streamline notetaking in straightforward cases, but the SNOMED CT suggestions were sometimes inaccurate or not specific enough for specialty needs. Other studies on AI documentation tools have similarly reported that user experience can be variable.^26^

From the patient’s perspective, the use of structured documentation was not a major concern. Consistent with previous studies, we found that patients prioritised communication quality and clinical outcomes over the method the clinician used to take notes.^27^

Accuracy, trustworthiness and preservation of privacy are of paramount importance for AI systems for healthcare. MiADE was hosted within the hospital infrastructure to ensure data security. The underlying algorithm (MedCAT^10^) uses dictionary matching combined with small neural network models. Unlike large language models, MiADE is not prone to hallucinations but is less effective at interpreting non-standard terms or nuanced contexts.

MiADE communicates with Epic using health data standards (Health Level 7 Clinical Document Architecture and SNOMED CT), and can easily be adapted to communicate with other EHRs.

### Recommendations

We suggest that buy-in from senior clinical leadership is essential for structured data recording to be considered an essential part of professional practice. Training and ongoing clinician engagement are also important to ensure that tools are as useful as possible.^24 25^

### Limitations

Our study yielded novel insights into the use of point of care NLP for structured data recording, but had several limitations.

First, participation was voluntary, relying on clinicians to contribute their time. This may have introduced selection bias; those with a particular interest in EHR may have been over-represented. Engagement among study participants was suboptimal. Sustained and longer term use would have enabled triangulation of qualitative insights with quantitative usage data.

Second, our MiADE implementation was limited by the specific EHR (Epic) and the existing NoteReader component. We were limited in our ability to control how the MiADE results were displayed to the user. For example, we had to prefix each diagnosis label with a number to ensure they appeared in the same order as in the text note (figure 1). As MiADE does not retain the analysed text, we were unable to evaluate its accuracy during the study itself, although we did so previously.^14^ Future versions of MiADE will be validated with additional samples of gold standard de-identified clinical notes.

Third, the quantitative evaluation was limited as there was no control group, so it is possible that the difference in diagnosis recording following MiADE go-live may have been due to other factors.

## Conclusion

MiADE was successfully implemented in a live clinical EHR and was associated with an increase in diagnosis recording in outpatients. Adoption among clinical users was mixed, shaped by differences in workflow, specialty requirements and individual perceptions of the system’s values. Future work should prioritise clinician co-design and better integration of structured documentation into clinical workflows.

## Supplementary material

10.1136/bmjhci-2025-101801 online supplemental file 1

10.1136/bmjhci-2025-101801 online supplemental file 2

10.1136/bmjhci-2025-101801 online supplemental file 3

10.1136/bmjhci-2025-101801 online supplemental file 4

## Data Availability

No data are available. All data relevant to the study are included in the article or uploaded as supplementary information.
