# Supplementary material for: Clinical evaluation of MiADE: a natural language processing system for assisting structured diagnosis recording at the point of care
Source: BMJ Health Care Inform. 2026 Feb 11;33(1):e101801. doi: 10.1136/bmjhci-2025-101801 (PMC12911726; doi:10.1136/bmjhci-2025-101801)
Supplement: online supplemental file 1 [file bmjhci-33-1-s001.pdf]

IRAS ID: 322887

CLINICIAN INTERVIEW TOPIC GUIDE

Feasibility study of ‘MiADE’ point of care natural language processing

Introduction

Interviewers will introduce themselves and verify consent for the interview.

Suggested phrases to open the interview:

- Thank you for speaking with me today and helping with this study.
- I have a list of topics that I want to address.
- Feel free to ask questions at any stage during the interview.
- I might make a few notes in case I want to come back to something later.

Interview prompts

- In your experience, how completely are problems, medications and allergies recorded in clinical practice?
  - Why do you think this is?
- Explore what influences their own behaviour in entering structured information
- How would you describe what MiADE does to one of your colleagues?
- In comparison to the ordinary Epic interface, how do you feel MiADE influences or changes the recording of structured information about problems, medications, allergies and medication intolerances?
  - Why do you think this is?
- How do you think MiADE could be improved?
- How do you think the Epic user interface for entering structured information could be improved?
- What else needs to change to improve the recording of structured information?

Interviewers will also ensure that clinicians have answered all the questions in the post-MiADE questionnaire, and if not, they will complete the questionnaire during the interview.

FUNDED BY NIHR | National Institute for Health and Care Research

UCL

MiADE

IRAS ID: 322887

PATIENT INTERVIEW TOPIC GUIDE

Feasibility study of 'MiADE' point of care natural language processing

Introduction

Interviewers will introduce themselves and verify consent for the interview.

Suggested phrases to open the interview:

- Thank you for speaking with me today and helping with this study.
- I have a list of topics that I want to address.
- Feel free to ask questions at any stage during the interview.
- I might make a few notes in case I want to come back to something later.

Interview prompts

- How did you feel about the way that the doctor interacted with the computer in your consultation?
- How did this compare with your experience of previous consultations in this hospital or elsewhere?
- Do you use the UCLH online patient portal (MyCare)? If so, I would like to ask you some questions about the information on medical diagnoses, allergies and medications recorded on MyCare. This information comes from specific places in the Epic electronic health record. MiADE is designed to make it easier for clinicians to fill in this information so that you can view it on MyCare.
    - Can you access information about your medical diagnoses, allergies and medications on MyCare?
    - How useful is this information?
    - How complete and accurate is this information?
- Open questions related to related to the importance of accuracy and completeness of information in the correct section of the record:
    - Benefits to treatment team (e.g. everyone knows where to find the information)
    - Barriers (e.g. it may take longer to enter information)
    - Benefits to patients (e.g. safer care, patients can see information on the patient portal)

FUNDED BY NIHR | National Institute for Health and Care Research

    - How else may structured information be useful? (e.g. direct care, research, monitoring health services)
- Would you be interested in being invited to future workshops or other activities to help improve electronic health records?
