# Supplementary material for: Clinical evaluation of MiADE: a natural language processing system for assisting structured diagnosis recording at the point of care
Source: BMJ Health Care Inform. 2026 Feb 11;33(1):e101801. doi: 10.1136/bmjhci-2025-101801 (PMC12911726; doi:10.1136/bmjhci-2025-101801)

Supplementary Figure 1: Study timeline

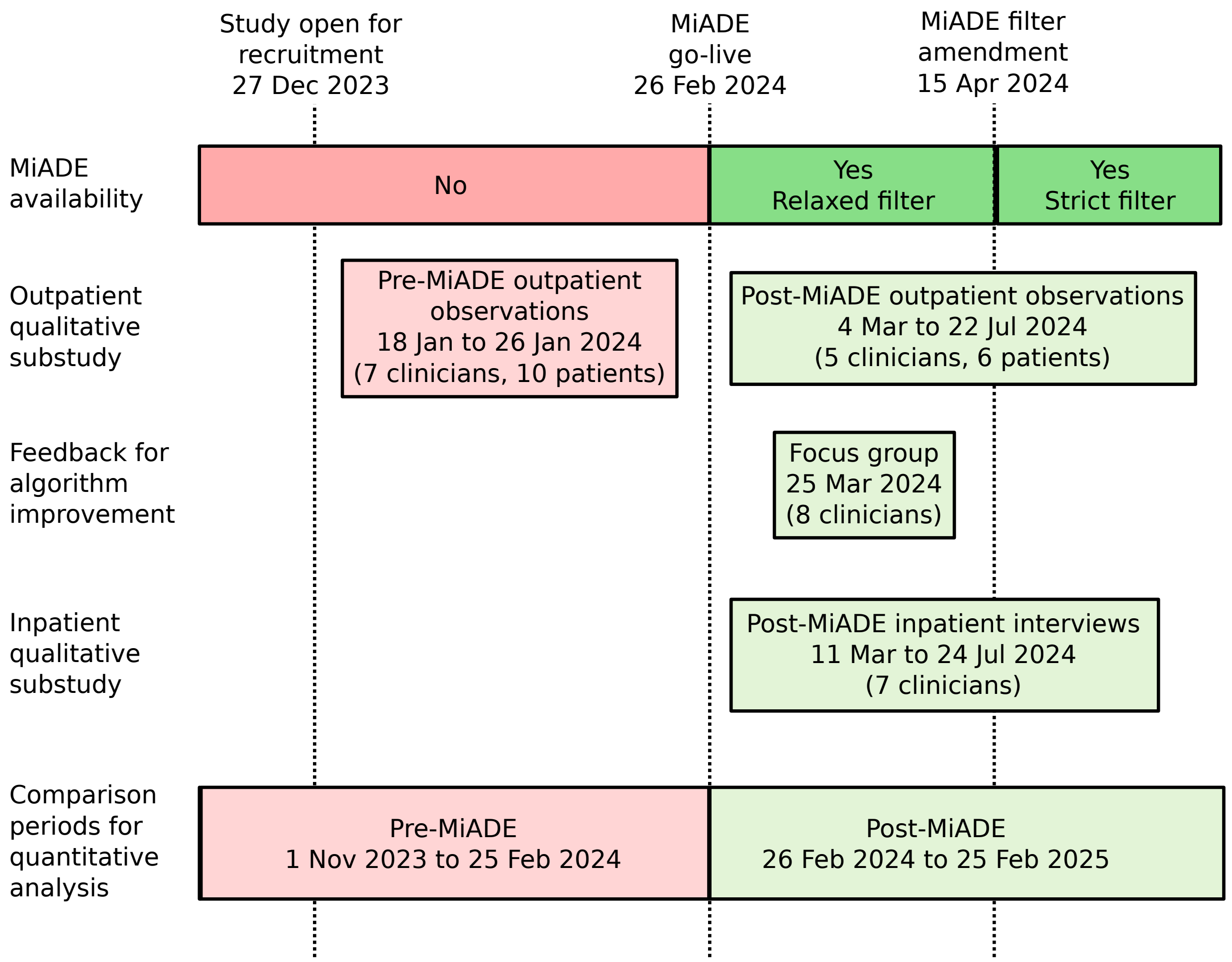

Supplementary Figure 2: Participant flow

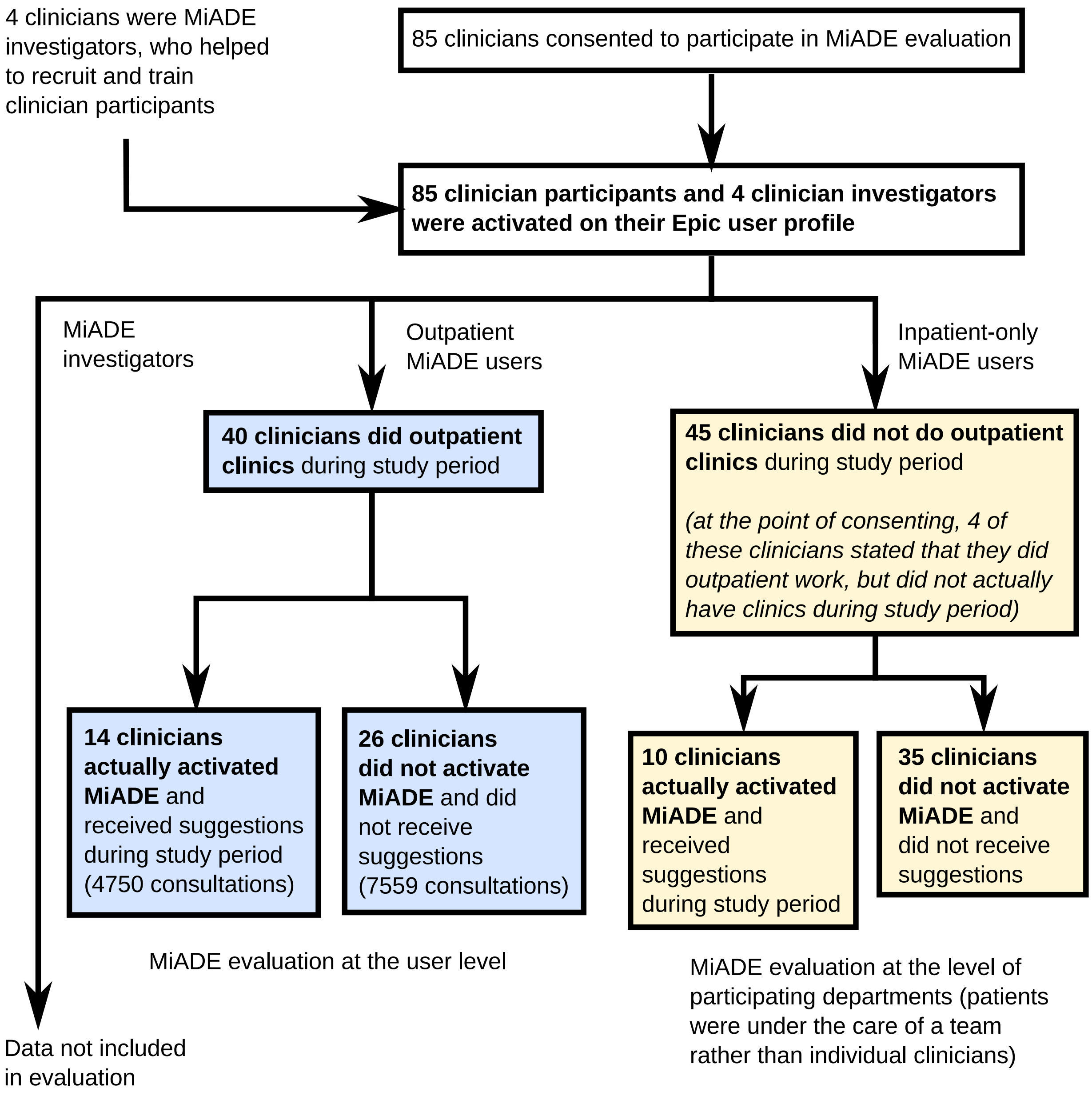

Supplementary Figure 3: MiADE suggestions accepted or rejected and and number of users over time

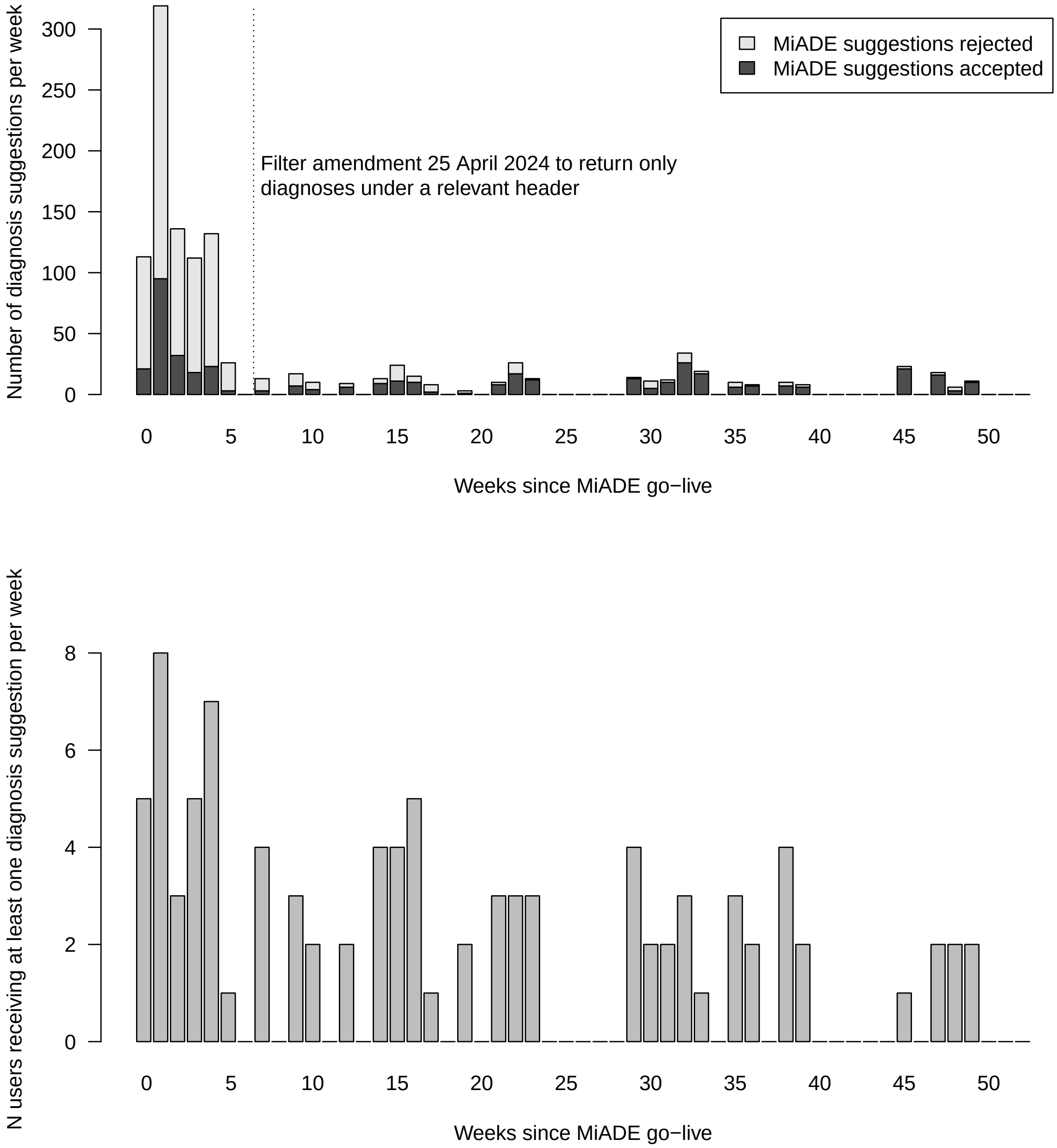

Supplement: online supplemental file 3 [file bmjhci-33-1-s003.pdf]
